# Pre-diagnostic 25-hydroxyvitamin D levels and subsite-specific colorectal cancer risk: a nested case–control study from the Norwegian Women and Cancer Study (NOWAC)

**DOI:** 10.1017/S0007114524003350

**Published:** 2025-02-14

**Authors:** Elise Marlen Paulsen, Tonje Bjørndal Braaten, Ilona Urbarova, Magritt Brustad

**Affiliations:** 1 Department of Community Medicine, University of Tromsø – The Arctic University of Norway, Tromsø, Norway; 2 The Public Dental Health Service Competence Centre of Northern Norway, Tromsø, Norway

**Keywords:** 25-hydroxyvitamin D, Colorectal cancer, Vitamin D, Proximal colon cancer, Distal colon cancer

## Abstract

Colorectal cancer (CRC), the third most common cancer globally, causes over 900 000 deaths annually. Although vitamin D is observed to have potential anti-carcinogenic properties, research findings on its preventable effect against CRC remain inconclusive. Notably, different subsites within the colon and rectum may be associated with distinct risk factors. While some studies have explored this relationship with circulating 25-hydroxyvitamin D (25(OH)D), the results remain contradictory. Our study employed a nested case–control design, involving 775 CRC cases matched with 775 cancer-free controls based on age, region of living and the time of blood sampling. The study was conducted within the Norwegian Women and Cancer post-genome cohort, which comprises approximately 50 000 women. We measured pre-diagnostic circulating plasma 25(OH)D status 5–13 years before diagnosis. Adjustment variables were based on self-administered questionnaires and included BMI, physical activity level, smoking, intake of processed meat, calcium, alcohol and fibre. An increase of 5 nmol/l in 25(OH)D reduced the risk of proximal colon cancer by 6 % (OR = 0·94, 95 % CI 0·89, 0·99). Furthermore, a sensitivity analysis revealed a 62 % increased risk among the women with 25(OH)D levels below 50 nmol/l compared with sufficient levels, ≥ 50 to < 75 nmol/l (OR = 1·62, 95 % CI 1·01, 2·61). No association was found with CRC, colon or distal colon cancer. We observed a subsite-specific association between 25(OH)D and CRC, highlighting the need for further investigation to elucidate the potential underlying mechanisms and clinical implications.

Vitamin D has an established role in bone mineralisation, essential for preventing rickets in children and osteomalacia in adults. It can be synthesised through skin exposure to sunlight or absorbed from dietary sources. Vitamin D_2_ primarily comes from plant sources such as mushrooms, whereas vitamin D_3_ comes from animal sources like fatty fish and egg yolks^(1)^. In the body, both vitamin D_2_ and vitamin D_3_ are converted to 25-hydroxyvitamin D (25(OH)D), which is the most widely used measurement of vitamin D status because it reflects both dietary intake and sun exposure.

In the kidneys, the enzyme CYP27B1 converts 25(OH)D to 1·25-hydroxyvitamin D (1·25(OH)D), the biologically active metabolite of vitamin D. This metabolite binds to the nuclear vitamin D receptor and is involved in the regulation of gene expression. Beyond its established role in maintaining calcium levels for bone mineralisation, emerging evidence suggests that vitamin D possesses additional extra skeletal functions, including anti-carcinogenic properties through the regulation of cellular processes such as proliferation, differentiation and apoptosis^(2,3)^. Vitamin D also influences inflammation, angiogenesis and immune modulation^(2–5)^. Additionally, it is suggested that vitamin D can alter the gut microbiome, thereby strengthening the intestinal barrier and enhancing the colon’s resistance to cancer development^(3,5)^.

Since the late 1980s, vitamin D has been investigated as a modifiable factor in the development of colorectal cancer (CRC)^(6)^. However, the results of these studies have been inconsistent, with the World Cancer Research Fund categorising the evidence of an association between vitamin D and CRC as limited^(7)^, which was supported by a scoping review conducted for the 2023 Nordic Nutrition recommendations^(1)^.

Different risk factors may be associated with cancer incidence in distinct colorectal subsites^(7–9)^. Some studies investigated these associations with a focus on dietary intake^(10–17)^ and others on blood measurements of circulating 25(OH)D^(18–23)^. The findings have been contradictory, as some studies found an association with distal colon and rectal cancer^(20,21)^, while others observed an association with proximal colon cancer^(18,19)^. Moreover, studies involving 25(OH)D have often been limited by a small number of cases^(20,21,23)^, and only one study focused exclusively on women^(20)^.

In a previously published study on vitamin D intake and the incidence of subsite-specific CRC risk within the Norwegian Women and Cancer (NOWAC) study, which involved 95 416 women and 1774 CRC cases, we found a 27 % reduced risk of proximal colon cancer with medium vitamin D intake (≥ 10 to < 20 µg/d) compared with low intake (< 10 µg/d), resulting in a hazard ratio of 0·73 (95 % CI 0·57, 0·94)^(10)^. No significant associations were found for CRC or other subsites. The study was limited to the estimation of dietary intake only and did not account for sun exposure. Therefore, we aimed to explore this association further by using blood measurements of 25(OH)D as exposure from the same women who took part in the mentioned NOWAC study. Specifically, we examined whether plasma 25(OH)D levels prior to diagnosis are associated with CRC or subsite-specific CRC risk in a nested case–control design using data from the NOWAC post-genome cohort study.

## Methods

### Study design

The NOWAC study is a prospective cohort study that includes around 170 000 women aged 30–70 years at enrolment^(24)^. Initiated in 1991, the study included women randomly selected from the national population register in Norway, all possessing a Norwegian personal identification number and born between 1927 and 1965^(24)^. Personal identification numbers are assigned to all individuals registered in the Norwegian population register and living in Norway, enabling them to follow these women until the incidence of cancer, event of death or emigration through national registries^(24)^.

Detailed self-administered questionnaires have been used one or more times among the women, encompassing a range of variables such as self-reported diseases, anthropometric measurements, reproductive history, socio-economic status, lifestyle and dietary habits^(24)^. Additionally, around 50 000 of the NOWAC women born 1943–1957 donated a blood sample between 2003 and 2006, which constitutes the NOWAC post-genome cohort study^(25)^. A more detailed description of the NOWAC study and the NOWAC post-genome cohort study has been published elsewhere^(24,25)^.

The NOWAC post-genome cohort study has received approval from the Norwegian Data Protection Authority (2002/2241) and the Regional Committees for Medical and Health Research Ethics (REK Nord 2003/01). This study was conducted according to the guidelines laid down in the Declaration of Helsinki, and all procedures involving human subjects were approved by REK Nord (application no. 520 423). Written informed consent was obtained from all women.

### Study sample

**Figure 1.**
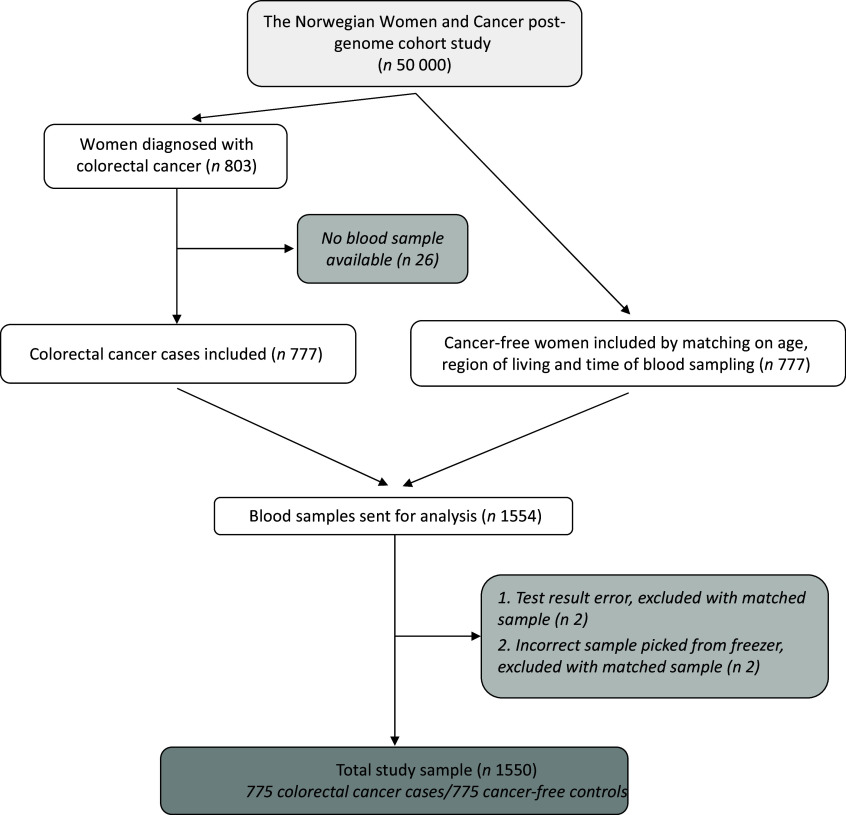
Flow chart of the inclusion process for the study.

For the present study, a 1:1 nested case–control design was employed using blood samples along with questionnaire data from the NOWAC post-genome cohort study. The mean time from completing the self-administered questionnaires to collecting blood samples was about 1 year, with a range of 5 months to 2·4 years. We utilised data from the Cancer Registry of Norway to identify all participants registered with an incidence of CRC by the end of 2018, totalling 803 cases among the 50 000 women followed in the cohort. However, blood samples were unavailable for twenty-six women, resulting in a total of 777 CRC cases. Each CRC case was matched with one cancer-free control based on age, region of living (north, middle, south-east or west of Norway) and time of blood sampling (within +/– 30 d). Consequently, 1554 women were initially included in the study population. Upon receiving the analysis result, one case was lost due to an error in the test results, and another had to be removed due to an incorrect retrieval from the freezer, resulting in the exclusion of their respective matched controls. This resulted in a total of 775 CRC cases and 775 cancer-free controls, comprising a total of 1550 women for the present study. A detailed flow chart of the inclusion process is presented in Fig. 1.

### Assessment of plasma 25-hydroxyvitamin D levels

In the NOWAC post-genome cohort study, the women received by mail a glass tube with citrate and needles together with an instruct to contact their local health centre for drawing the blood samples^(25)^. The samples were dispatched via prepaid high-priority overnight mail to the Department of Community Medicine at UiT The Arctic University of Tromsø, Norway^(25)^. Upon arrival, the blood samples were centrifuged, the buffy coat was separated and the samples were stored in 2 ml cryotubes at –80°C, until the plasma was shipped to the Department of Laboratory Medicine, University Hospital of North Norway for analysis. A total of 80 µl of plasma was analysed for each of the 1550 samples across nineteen plates. A detailed description of the laboratory’s analysis method can be found elsewhere^(26)^.

The laboratory is accredited for vitamin D analyses in accordance with the National Standard European Norm International Organization for Standardization 15 189 and participates in the Vitamin D External Quality Assessment Scheme’s ring test programme (External Quality Assessment) four times a year.

Shortly, 25-hydroxyvitamin D2 (25(OH)D2) and 25-hydroxyvitamin D3 (25(OH)D3) levels were analysed using liquid chromatography with tandem MS on ninety-six deep-well plates, incorporating two controls (MassCheck® 25-OH-Vitamin D3/D2 and 3-epi-25-OH-Vitamin D3 Serum Controls, Chromsystems) and six standards both at the beginning and the end of each plate to ensure quality assurance. The six standards consisted of one standard sample that was serially diluted in six stages, each stage halving the concentration of the previous one. Furthermore, to ensure the precision and reliability of the analyses, the laboratory adheres to strict quality criteria, one of which is that the correlation coefficient (R^2^) for the standard curve must exceed 0·99 before results are reported.

Given that the cases and controls were not consistently processed on the same plate, there was a potential risk of introducing bias due to plate-specific variability in the 25(OH)D measurements. To address this issue, we standardised the 25(OH)D measurements. A CV was calculated for each standard per plate by comparing the actual measured value to the expected concentration. Further, the mean of the CV per plate was calculated, multiplied by each 25(OH)D measurement per plate, and this value was subtracted from each 25(OH)D measurement on that plate (25(OH)D - 25(OH)D * mean CV)). The standardised values were subsequently used for further analysis. In the statistical analysis, we merged 25(OH)D2 and 25(OH)D3 into one variable, hereafter referred to as 25(OH)D.

### Covariates

**Figure 2.**
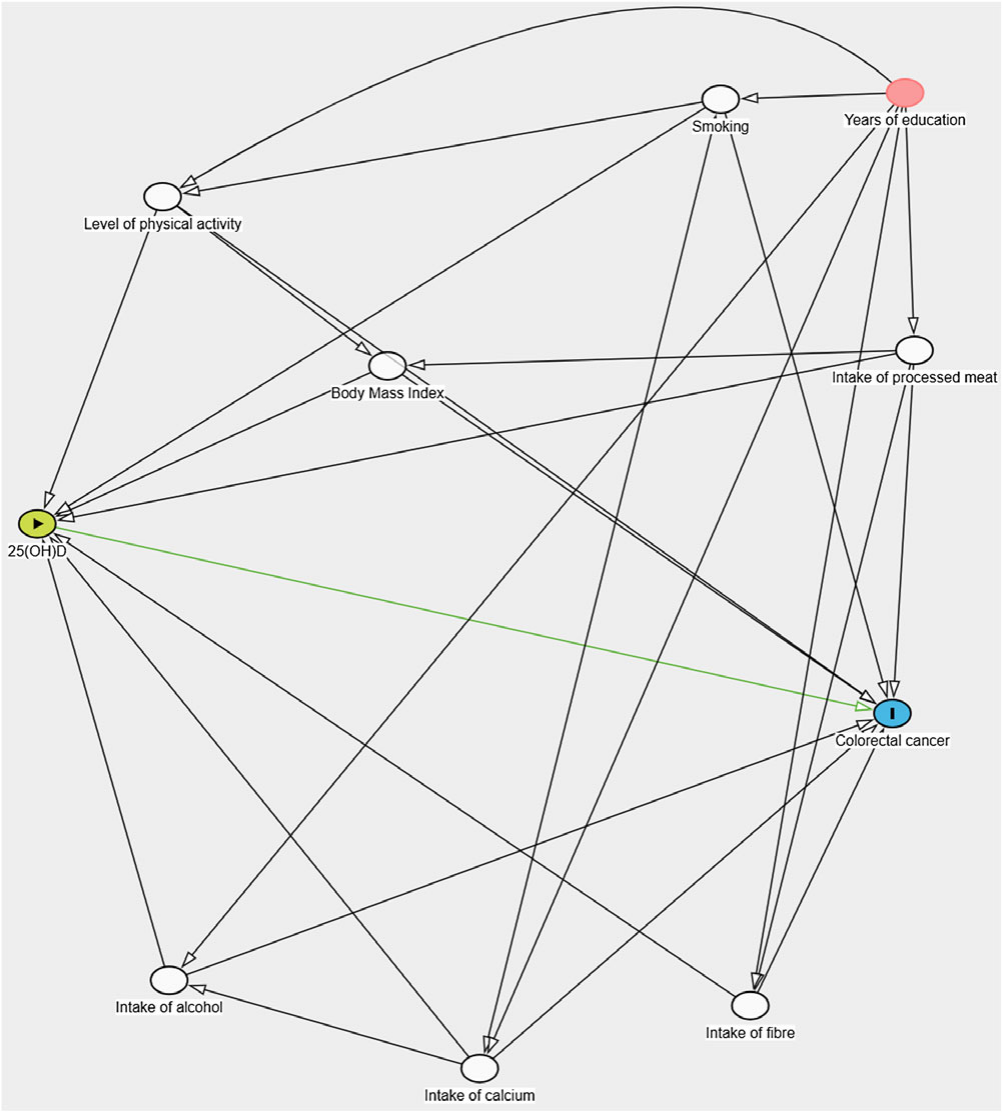
Directed acyclic graph illustrating the association between 25-hydroxyvitamin D (green node) and colorectal cancer (blue node). White nodes represent confounding variables in the pathway between 25-hydroxyvitamin D and colorectal cancer. The red node indicates an ascendant pathway influenced by lifestyle and dietary factors, which is closed by adjusting for these factors.

We identified potential confounders by reviewing the literature on 25(OH)D and CRC and employed a directed acyclic graph prior to the analysis (Fig. 2). The following variables were identified as covariates: level of physical activity (PA), smoking status, BMI, processed meat intake, alcohol intake, fibre intake and calcium intake. Age, retrieved from the National Registry of Norway, was acknowledged as a potential confounding variable and has already been controlled for by matching. Considering that 25(OH)D levels fluctuate with the season, and the likelihood of decreased sunlight exposure at higher latitudes, we controlled for this by matching the women based on time of blood sampling and region of living.

The level of PA was assessed on a score from 1 to 10, with 1 indicating very low activity and 10 indicating very high activity^(27)^. The reporting of PA has previously been validated^(27)^. In the analysis, we classified PA into three categories: low (1–4), medium (5–6) and high (7–10). Smoking status was categorised into three groups: never smoker, former smoker and current smoker. BMI, calculated as weight in kilograms divided by the square of height in metres, was classified according to the thresholds of the WHO and based on previously validated self-reported height and weight^(28)^: underweight (< 18·5), normal weight (≥ 18·5 to < 25), overweight (≥ 25 to < 30) and obese (≥ 30). Processed meat intake, alcohol intake, fibre intake and calcium intake were derived from the responses to previously validated self-administered FFQ^(29)^. These FFQ assess food consumption over the last year. Nutrient content in reported intake has been calculated using the Norwegian Food Composition Database^(30)^, and reported frequencies were converted into grams by using the Norwegian Weight and Measurement Table^(31)^. Processed meat intake was calculated from the combination of the reported consumption of meatballs, cold cuts and sausages.

Due to the right-skewed distribution of processed meat, alcohol and calcium intake, we examined whether treating these variables as continuous or categorised would affect their confounding effect on the relationship between 25(OH)D and CRC risk. Processed meat intake was investigated by dividing the variable into tertiles, compared with using it as a continuous variable. Additionally, alcohol intake was examined and categorised into no consumption (≤ 0 g/d), moderate (> 0 to < 10 g/d) and high (≥ 10 g/d). Calcium intake was examined categorising it into two groups, divided by the median, above and below 669 mg/d. However, the OR remained unchanged, and we retained all the FFQ variables as continuous in the statistical model. In addition, we tested if excluding women who developed cancer within 1 year after the blood sample draw (*n* 26) and those with a total energy intake below 500 kcal/d (*n* 4) would change the OR of the analysis. As this was not the case, we decided to retain them in the dataset. Lastly, none of the women consumed more than 3500 kcal/d.

### Outcome

CRC cases were identified through linkage to the Cancer Registry of Norway and classified using the International Classification of Diseases, Tenth Revision (ICD-10), codes C18–C18·9, and International Classification of Diseases for Oncology, Third Edition (ICD-O-3), topography codes C199 and C209. In the statistical analysis, CRC encompasses all cases occurring in the colon or rectum, including C199, which represents cancer in the rectosigmoid region, where the colon and rectum meet. Colon cancer includes cases located in proximal colon (C18·0–C18·4), distal colon (C18·5–C18·7) and those classified under codes C18, C18·8 and C18·9. The last three codes represent cancers located in the caecum with ileocecal valve, overlapping lesion of the colon or colon unspecified, respectively; therefore, we did not categorise them as proximal or distal colon cancer. Proximal and distal colon cancers were in addition, analysed individually. For clarity, cancers occurring to the right of, or proximal to, the splenic flexure are categorised as proximal colon cancer, while cancers occurring to the left of, or distal to, the splenic flexure are categorised as distal colon cancer. Rectal cancer was classified under C209.

### Statistical methods

Prior to the analysis, missing values were replaced using multiple imputation with the chained equation approach, assuming the data were missing at random. Each missing value was imputed across twenty datasets. Missing data were imputed for the following variables: BMI, with a total of fifty missing values, comprising twenty-three from height and forty-nine on weight; PA, with a total of ninety-five missing values; years of education, with eighty-four missing values; smoking status with twenty missing values; and intake of alcohol with thirty-two missing values. All other variables included in the analysis model were also included in the imputation model.

To test for differences across 25(OH)D levels on a nominal scale, we used the *χ* ^2^ statistic for categorical variables. For continuous variables, we used one-way ANOVA or Kruskal–Wallis’s test when equal variances were not assumed. The distribution of variables at baseline is shown as means with standard deviations (sd) or percentages in Table 1. The risk of CRC and colorectal subsites were investigated using 25(OH)D on the continuous scale and grouping the variable into four categories, based on the Nordic Nutrition recommendations: deficiency (≤ 30 nmol/l), insufficiency (> 30 to < 50 nmol/l), sufficiency (≥ 50 to < 75 nmol/l) and high (≥ 75 nmol/l) status of 25(OH)D, with sufficiency serving as the reference category^(1)^.

**Table 1.** Baseline descriptives of the study sample (*n* 1550) by 25-hydroxyvitamin D plasma levels in the Norwegian Women and Cancer post-genome cohort (2003–2006) (Numbers and percentages; mean values and standard deviations)

	Grouped 25-hydroxyvitamin D status, measured in nmol/l*								
	Deficiency ≤ 30		Insufficiency > 30 to < 50		Sufficiency ≥ 50 to < 75		High ≥ 75		*P*
	*n*	%	*n*	%	*n*	%	*n*	%	
Number of participants, *n* (%)	120	8	558	36	718	46	154	10	0·21
Number of colorectal cancer cases, *n* (%)	67	9	287	37	340	44	81	10	
Number of cancer-free controls, *n* (%)	53	7	271	35	378	49	73	9	
	Mean	sd	Mean	sd	Mean	sd	Mean	sd	
Age in years, mean	56		55		55		55		0·26
25(OH)D levels in nmol/l, mean (sd)	23·8	4·8	41·2	5·6	60·3	6·8	85·9	9·6	< 0·001
Intake of fibre (g/d), mean (sd)	20	7	22	7	22	7	22	7	0·012
Intake of calcium (g/d), mean (sd)	681	301	704	284	719	299	706	257	0·52
Intake of processed meat (g/d), mean (sd)**	31	19	31	20	30	19	28	19	0·30
Intake of alcohol (g/d), mean (sd)†	4	5	4	5	4	5	6	7	0·011
Energy intake (kcal/d), mean (sd)	1609	433	1673	458	1727	475	1740	478	0·02
	*n*	%	*n*	%	*n*	%	*n*	%	
BMI/kg/m^2^, *n* (%)‡									
≤ 18·5	1	1	3	1	7	1	3	2	< 0·001
> 18·5 to < 25	50	42	235	44	407	58	102	68	
≥ 25 to < 30	35	29	193	36	217	31	40	27	
≥ 30	33	28	101	19	68	10	5	3	
Region of living, *n* (%)									
South-Eastern Norway	55	46	230	41	351	49	76	49	0·05
Western Norway	22	18	110	20	128	18	38	25	
Central Norway	12	10	75	14	79	11	18	12	
Northern Norway	31	26	143	26	160	22	22	14	
Level of physical activity, *n* (%)§									
Low	41	38	152	29	115	17	26	18	< 0·001
Medium	39	36	225	43	285	42	60	41	
High	28	26	147	28	278	41	59	41	
Educational years, *n* (%)ǁ									
≤ 9 years	42	37	100	19	138	20	26	18	< 0·001
10–12 years	47	41	209	40	225	33	56	39	
13–16 years	20	17	144	27	216	32	37	25	
≥ 17 years	6	5	73	14	101	15	26	18	
Smoking, *n* (%)¶									
Never	29	25	203	37	278	39	56	37	< 0·001
Former	44	37	204	37	298	42	67	44	
Current	45	38	146	26	131	19	29	19	

nmol/l, nanomole per litre; 25(OH)D, 25-hydroxyvitamin D; g/d, gram per d; kg/m^2^, kilograms divided by height in meters^2^.

* 25-Hydroxyvitamin D is derived from the combination of D2 and D3, measured in plasma.

† Thirty-two missing values.

‡ Fifty missing values.

§ Ninety-five missing values.

ǁ Eighty-four missing values.

¶ Twenty missing values.

** Processed meat = meatballs, sausages and cold cuts.

We employed conditional logistic regression based on the previously described matching criteria as mentioned above (age, region of living and time of blood sampling) and used two models to explore the association: one unadjusted (crude) model and one multivariable model adjusted for PA, BMI, smoking, processed meat intake, alcohol intake, fibre intake and calcium intake. We performed analyses both with multiple imputed values (Table 2, *n* 1550) and one with only complete cases (online Supplementary Table 1, *n* 1250 for multivariable model). Due to the low number of women in the deficient and insufficient categories, we conducted a sensitivity analysis by combining these two lowest categories, representing 25(OH)D status below 50 nmol/l, and compared them with the sufficient (≥ 50 to < 75 nmol/l) and high 25(OH)D status (≥ 75 nmol/l) categories using the multiple imputation model (online Supplementary Table 2).

**Table 2.** Risk of colorectal cancer, colon cancer, proximal colon cancer, distal colon cancer and rectal cancer according to plasma 25-hydroxyvitamin D levels. A nested case–control study (*n* 1550) from the Norwegian Women and Cancer post-genome cohort (2003–2006) (Odds ratios and 95 % confidence intervals)

		Matched by age, region of living and time of blood sampling			Multivariable analyses*		
	*n* (cases/controls)	OR	95 % CI	*P*	OR	95 % CI	*P*
Colorectal cancer							
Continuous (5 nmol/l increase)	775/775	0·97	0·94, 1·00	0·10	0·97	0·94, 1·00	0·09
* *Grouped							
≤ 30 nmol/l	67/53	1·44	0·96, 2·16	0·07	1·43	0·94, 2·16	0·09
> 30 to < 50 nmol/l	289/274	1·19	0·95, 1·51	0·12	1·22	0·96, 1·55	0·11
≥ 50 to < 75 nmol/l (reference value)	338/375	1·00			1·00		
≥ 75 nmol/l	81/73	1·22	0·85, 1·74	0·28	1·19	0·82, 1·72	0·35
Colon cancer							
Continuous (5 nmol/l increase)	525/525	0·97	0·93, 1·01	0·14	0·97	0·93, 1·01	0·14
* *Grouped							
≤ 30 nmol/l	49/39	1·40	0·87, 2·24	0·17	1·36	0·83, 2·22	0·23
> 30 to < 50 nmol/l	193/187	1·14	0·86, 1·51	0·37	1·15	0·86, 1·54	0·33
≥ 50 to < 75 nmol/l) (reference value)	231/251	1·00			1·00		
≥ 75 nmol/l	52/48	1·17	0·75, 1·84	0·49	1·16	0·73, 1·84	0·52
Proximal colon cancer							
Continuous (5 nmol/l increase)	321/321	0·95	0·90, 1·00	0·05	0·94	0·89, 0·99	0·03
* *Grouped							
≤ 30 nmol/l	30/25	1·28	0·71, 2·30	0·41	1·33	0·71, 2·49	0·37
> 30 to < 50 nmol/l	119/112	1·13	0·80, 1·59	0·50	1·17	0·82, 1·67	0·39
≥ 50 to < 75 nmol/l (reference value)	147/154	1·00			1·00		
≥ 75 nmol/l	25/30	0·83	0·45, 1·54	0·56	0·81	0·43, 1·53	0·51
Distal colon cancer							
Continuous (5 nmol/l increase)	198/198	1·00	0·93, 1·07	0·96	1·01	0·94, 1·08	0·80
* *Grouped							
≤ 30 nmol/l	19/13	1·83	0·80, 4·17	0·15	1·55	0·65, 3·67	0·32
> 30 to < 50 nmol/l	72/72	1·23	0·75, 2·02	0·41	1·23	0·73, 2·07	0·44
≥ 50 to < 75 nmol/l (reference value)	82/96	1·00			1·00		
≥ 75 nmol/l	25/17	1·74	0·87, 3·48	0·12	1·71	0·83, 3·52	0·14
Rectal cancer							
Continuous (5 nmol/l increase)	221/221	0·98	0·92, 1·04	0·50	0·98	0·91, 1·04	0·47
* *Grouped							
≤ 30 nmol/l	16/12	1·71	0·75, 3·91	0·20	1·83	0·77, 4·38	0·16
> 30 to < 50 nmol/l	86/76	1·43	0·91, 2·24	0·12	1·60	0·98, 2·59	0·06
≥ 50 to < 75 nmol/l (reference value)	90/111	1·00			1·00		
≥ 75 nmol/l	29/22	1·55	0·82, 2·92	0·18	1·61	0·81, 3·19	0·17

* Adjusted for age, region of living and time of blood sampling (all through matching), with additional adjustments for physical activity, BMI, smoking, processed meat, alcohol, calcium and fibre intake.

The reported results are presented as odds ratios (OR) and 95 % confidence intervals (CI). A *P*-value of less than 0·05 was considered statistically significant. The statistical analysis was carried out using Stata/MP 17·0.

## Results

### Baseline descriptives

From all 1550 study participants, 46 % (*n* 712) women were living in the South-Eastern Norway, 19 % (*n* 298) in the Western Norway, 12 % (*n* 184) in the Central Norway and 23 % (*n* 356) in the Northern Norway (Table 1). Among the 775 CRC cases, 29 were diagnosed with cancer in the rectosigmoid region, 525 had colon cancer and 221 had rectal cancer. Of those with colon cancer, 321 had proximal colon cancer, 198 had distal colon cancer and 6 were classified under the codes C18, C18·8 or C18·9. The average time from blood sampling to a CRC diagnosis was 5–13 years, with an average of 9 years.

Among the CRC cases, approximately 9 % were classified as 25(OH)D deficient, 37 % as insufficient, 44 % as sufficient and 10 % as having high status, and for cancer-free controls, the respective percentages were 7, 35, 49 and 9 %.

The mean fibre intake increased from 20 g/d in the insufficient to 22 g/d in the high-status group of 25(OH)D (*P*-value = 0·012; Table 1). Similarly, mean alcohol consumption increased from 4 g/d (sd = 5) in the deficient to 6 g/d (sd = 7) in the high-status group of 25(OH)D (*P*-value = 0·011; Table 1). Furthermore, 25(OH)D levels increased with increasing energy intake from 1609 kcal/d (sd = 433) in the deficient group to 1740 kcal/d (sd = 478) in the high-status group (*P*-value = 0·019; Table 1).

A greater prevalence of obesity (BMI=≥ 30) was observed in the deficient 25(OH)D group (28 %) compared with the high-status group (3 %). In contrast, a normal BMI (≥ 18·5 to < 25) was more prevalent in the sufficient (58 %) and high-status (68 %) groups than in the deficient 25(OH)D group (42 %, *P*-value < 0·001; Table 1).

Among those with deficient 25(OH)D status, 38 % of the women reported low PA, and 26 % reported high levels of PA. Conversely, in the high 25(OH)D status group, only 18 % reported low PA, while 41 % reported high levels of PA (*P*-value < 0·001; Table 1). Lastly, there was a higher proportion of current smokers in the deficient group than in the sufficient and high-status groups, at 38 % compared with 19 %, respectively (*P*-value < 0·001; Table 1).

### Regression analysis and risk for total CRC and its subsites

Results from the conditional regression analysis are shown in Table 2. A significantly reduced risk of proximal colon cancer was observed with a 5 nmol/l increase in 25(OH)D levels between cases and cancer-free controls (multivariable analysis; OR = 0·94, 95 % CI 0·89, 0·99). No association between a 5 nmol/l increase in 25(OH)D levels and the incidence of CRC, colon cancer, distal colon cancer or rectal cancer was observed.

The categorical analysis revealed no significant associations between deficient (≤ 30 nmol/l), insufficient (> 30 to < 50 nmol/l) and high 25(OH)D status (≥ 75 nmol/l) compared with sufficient 25(OH)D status (≥ 50 to < 75 nmol/l), among cases and cancer-free controls for CRC, colon, proximal colon cancer, distal colon cancer or rectal cancer (Table 2). However, in the sensitivity analysis, we observed a 62 % increased risk among women with 25(OH)D status below 50 nmol/l compared with sufficient levels (≥ 50 to < 75 nmol/l), among rectal cancer cases and cancer-free controls (multivariable analysis; OR = 1·62, 95 % CI 1·01, 2·61; online Supplementary Table 2).

Minimal confounding was observed between the crude and the multivariable models. Furthermore, no substantial differences were observed between the complete case analysis (online Supplementary Table 1) and the multiple imputed analysis (Table 2).

## Discussion

In this nested case–control study, no significant associations were observed between plasma 25(OH)D levels, measured on a continuous scale or categorised into groups, and the risk of CRC, colon cancer or distal colon cancer. Notably, we found that for a 5 nmol/l increase in 25(OH)D levels, the risk of proximal colon cancer decreased by 6 %. Additionally, in the sensitivity analysis, we found a 62 % increased risk among women with 25(OH)D status below 50 nmol/l compared with sufficient levels (≥ 50 to < 75 nmol/l), among rectal cancer cases and cancer-free controls.

Our findings indicate a reduced risk of proximal colon cancer with increasing 25(OH)D levels, aligning with McCullough *et al.* ^(18)^, who, when pooling data from seventeen cohort studies, observed a stronger association with proximal than distal colon cancer. They reported a relative risk of 0·81 (95 % CI 0·73, 0·90) for proximal colon cancer and a relative risk = 0·93 (95 % CI 0·81, 1·08) for distal colon cancer per 25 nmol/l increase in 25(OH)D. In the Prostate, Lung, Colorectal, and Ovarian Cancer Screening Trial cohort, Weinstein *et al.* ^(19)^ observed a reduced risk for proximal but not for distal colon cancer using season-specific quartiles, with an OR of 0·49 (95 % CI 0·30, 0·82) for the highest *v*. lowest quartile of 25(OH)D, based on 300 proximal and 119 distal colon cancer cases. Additionally, the linear trend was significant for proximal colon cancer (*P*= 0·01), but not for distal colon cancer (*P*= 0·23). Conversely, Feskanich *et al.* ^(20)^ observed a potentially stronger association between 25(OH)D levels and the risk of distal colon and rectal cancer, but not proximal colon cancer, in a nested case–control study including 193 CRC cases from the Nurses’ Health Study. However, the limited number of cases in both proximal (*n* 78) and distal (*n* 61) colon constrained the strength of the associations, leading to a combined analysis of distal colon and rectal cancer that revealed a significant linear trend (*P*= 0·02).

Tangrea *et al.* ^(21)^ observed a similar association with combined distal colon and rectal cancer, but no significant association for each subsite individually, limited by the small number of cases: forty-three proximal colon and forty-eight distal colon cancer cases. However, their analysis was conducted exclusively on men. Notably, proximal colon cancer occurs more frequently in women, whereas distal colon cancer is more common in men^(8,9)^. Given that our study targets women, we are more likely to observe an association with proximal colon cancer. Lastly, another nested case–control study based on males from the Health Professionals Follow-up Study^(23)^ pooled results with the Nurses’ Health Study. However, they revealed no significant differential association between proximal and distal colon cancer, emphasising the limited number of participants as a potential constraint on the robustness of the findings^(23)^.

In our previously published study on vitamin D intake and the incidence of subsite-specific CRC risk, we found a 27 % reduced risk of proximal colon cancer with medium vitamin D intake (≥ 10 to < 20 µg/d) compared with low intake (< 10 µg/d)^(10)^. Notably, 72 % of the women consumed less than the recommended daily intake of 10 µg/d. This intake calculation was based solely on dietary sources, thus excluding synthesis from sun exposure, leaving the actual 25(OH)D blood levels among the women unknown. In the present study, more than half of the women had sufficient 25(OH)D levels, with almost equal mean levels among cases and controls. The narrow range of 25(OH)D status makes it challenging to detect potential associations between 25(OH)D and CRC or CRC subsites.

Our previous study on CRC risk by vitamin D intake^(10)^ revealed a stronger association than the present study on circulating 25(OH)D. Some of the ingested vitamin D passes through the small intestine without being absorbed, and the remainder transfers to the colon, initially entering the proximal part, where it becomes available for bacterial fermentation. Studies have found that vitamin D can alter the gut microbiome, thereby strengthening the intestinal barrier and enhancing the colon’s resistance to cancer development^(3)^. Thus, the effects of vitamin D may not solely depend on the levels of vitamin D metabolites in blood but also on the direct interaction with the microbiome in the colon^(5)^. This may suggest why we did not find the same risk reduction in the present study using blood levels of 25(OH)D as the exposure.

We observed a distinct association of proximal colon cancer with blood levels of 25(OH)D compared with other colorectal subsites. A 5 nmol/l increase in 25(OH)D significantly reduced cancer risk by 6 %. This is supported by the categorical analysis where the OR decrease as the 25(OH)D levels increase across categories, though not significant. Notably, this subsite is the only site where the OR falls below 1 (OR = 0·89) in the high 25(OH)D category. In contrast, although not significant, other subsites indicate an increased risk in the high 25(OH)D category, with no beneficial effect from levels exceeding the sufficient 25(OH)D category. Employing continuous analysis allows us to examine potential dose–response relationships. On the other hand, the categorical approach enables the identification of non-linear relationships. For rectal cancer, the continuous approach showed no statistically significant effect. However, the sensitivity analysis revealed a 62 % increased risk among those with lower levels of 25(OH)D (< 50 nmol/l) compared with sufficient levels (≥ 50 to < 75). This indicates that the low number of cases and controls in the original analysis (Table 2) likely constrained the possibility of a significant finding.

CRC exhibits diverse molecular features, including microsatellite instability, chromosomal instability, CpG island methylator phenotype and mutations in genes like KRAS and TP53^(32)^. Microsatellite instability is more prevalent in proximal colon cancer, while chromosomal instability is common in the distal colon^(9,32)^, suggesting that the role of vitamin D may vary based on the disease origin. The conversion of 25(OH)D to its active form in immune cells suggests an intracrine system that could influence cancer development through immunomodulatory actions^(2,4)^. Given the link between inflammatory bowel diseases and increased CRC risk, the role of vitamin D in immune modulation may be crucial in reducing CRC risk and warrants further research^(2,4,5,33,34)^.

One of the methodological strengths of the NOWAC study is the utilisation of random selection of women from the national population register, which minimises selection bias. The overall response rate was 57 %^(35)^, with demographic disparities evident among the responders. Those who responded tended to have a higher educational level and were predominantly of younger age groups^(35)^. Despite these disparities, there is no evidence to suggest that the risk of CRC differs between respondents and non-respondents. Thus, we believe the study findings remain generalisable^(24,35)^. Compared with studies on vitamin D intake, measuring 25(OH)D provides a more reliable assessment, as it covers both dietary intake and cutaneous vitamin D production, avoiding biases related to reporting, memory and recall. Although 25(OH)D was measured only once for each participant, thus questioning to what degree it reflects long-term status, evidence supports the sufficiency of a single measurement of vitamin D for studying future diseases^(36)^. The number of women in each category of 25(OH)D may be too limited to establish an association. However, in the continuous analysis, our study benefits from a large sample size compared with previous studies, particularly for each subsite. Additionally, a significant strength of our study is the comprehensive inclusion of every case from the 50 000 women involved in the NOWAC post-genome cohort.

The questionnaire and the blood samples were not collected simultaneously, which could result in residual confounding.

Although twenty-six women developed cancer within 1 year after their blood sample was taken, suggesting that cancer progression might have already started, excluding these cases did not change the results.

We imputed missing values to avoid attenuating the statistical strength^(37)^. Comparing the complete case analysis and the imputed analysis, the results remain the same, which strengthens our findings. The finding on proximal colon cancer was borderline significant in the unadjusted analysis but not in the multiple adjusted analysis; however, we lost fifty-five cases without imputation. The association became significant when we kept those cases in the imputed dataset.

These findings suggest that the relationship between 25(OH)D and CRC may be specific to certain subsites within the colon and rectum. Particularly, the association with proximal colon cancer and rectal cancer warrants further investigation to elucidate the underlying mechanisms and potential clinical implications.

### Conclusion

Increased pre-diagnostic 25(OH)D levels were associated with a reduced risk of proximal colon cancer, while having low levels of 25(OH)D was associated with an increased risk of rectal cancer. No significant association was observed between 25(OH)D and the risk of CRC, colon or distal colon cancer.

## Supporting information

Paulsen et al. supplementary material Paulsen et al. supplementary material

## Supplementary material

For supplementary material/s referred to in this article, please visit https://doi.org/10.1017/S0007114524003350.
