# Supplementary material for: Pre-diagnostic 25-hydroxyvitamin D levels and subsite-specific colorectal cancer risk: a nested case–control study from the Norwegian Women and Cancer Study (NOWAC)
Source: Br J Nutr. 2025 Jan 3;133(3):363–71. doi: 10.1017/S0007114524003350 (PMC11946035; doi:10.1017/S0007114524003350)
Supplement: Paulsen et al. supplementary material [file S0007114524003350sup001.docx]

**Supplementary Material**

**Supplementary Table 1** Complete case analysis of the risk of colorectal cancer, colon cancer, proximal colon cancer, distal colon cancer, and rectal cancer according to plasma 25-Hydroxyvitamin D levels. A nested case-control study (n=1550) from the Norwegian Women and Cancer post-genome cohort (2003-2006).

|  |  | Matched by age, region of living, and time of blood sampling | | | |  | Multivariable analyses* | | | |  |
| --- | --- | --- | --- | --- | --- | --- | --- | --- | --- | --- | --- |
|  |  | n (cases / controls) | OR | 95% CI | *p* |  | n (cases / controls) | OR | 95% CI | *P* |  |
| **Colorectal cancer** |  |  |  |  |  |  |  |  |  |  |  |
| Continuous (5 nmol/L increase) |  | 775/775 | 0.97 | 0.94-1.00 | 0.10 |  | 625/625 | 0.98 | 0.95-1.02 | 0.38 |  |
| *Grouped* |  |  |  |  |  |  |  |  |  |  |  |
| ≤30 nmol/L |  | 67/53 | 1.44 | 0.96-2.16 | 0.07 |  | 47/47 | 1.03 | 0.65-1.65 | 0.90 |  |
| >30 to <50 nmol/L |  | 289/274 | 1.19 | 0.95-1.51 | 0.14 |  | 232/217 | 1.17 | 0.90-1.53 | 0.24 |  |
| ≥50 to <75 nmol/L (reference value) |  | 338/375 | 1.00 |  |  |  | 278/301 | 1.00 |  |  |  |
| ≥75 nmol/L |  | 81/73 | 1.22 | 0.85-1.74 | 0.29 |  | 68/60 | 1.21 | 0.80-1.82 | 0.37 |  |
| **Colon cancer** |  |  |  |  |  |  |  |  |  |  |  |
| Continuous (5 nmol/L increase) |  | 525/525 | 0.97 | 0.93-1.01 | 0.14 |  | 429/429 | 0.98 | 0.93-1.02 | 0.33 |  |
| *Grouped* |  |  |  |  |  |  |  |  |  |  |  |
| ≤30 nmol/L |  | 49/39 | 1.40 | 0.87-2.24 | 0.17 |  | 35/33 | 1.01 | 0.58-1.77 | 0.97 |  |
| >30 to <50 nmol/L |  | 193/187 | 1.14 | 0.86-1.51 | 0.37 |  | 154/151 | 1.08 | 0.78-1.49 | 0.65 |  |
| ≥50 to <75 nmol/L (reference value) |  | 231/251 | 1.00 |  |  |  | 195/203 | 1.00 |  |  |  |
| ≥75 nmol/L |  | 52/48 | 1.17 | 0.75-1.84 | 0.49 |  | 45/42 | 1.14 | 0.69-1.89 | 0.61 |  |
| **Proximal colon cancer** |  |  |  |  |  |  |  |  |  |  |  |
| Continuous (5 nmol/L increase) |  | 321/321 | 0.95 | 0.90-1.00 | 0.05 |  | 266/266 | 0.96 | 0.90-1.02 | 0.17 |  |
| *Grouped* |  |  |  |  |  |  |  |  |  |  |  |
| ≤30 nmol/L |  | 30/25 | 1.28 | 0.72-2.32 | 0.41 |  | 22/22 | 0.94 | 0.46-1.93 | 0.87 |  |
| >30 to <50 nmol/L |  | 119/112 | 1.13 | 0.81-1.61 | 0.50 |  | 95/95 | 0.96 | 0.64-1.43 | 0.83 |  |
| ≥50 to <75 nmol/L (reference value) |  | 147/154 | 1.00 |  |  |  | 126/121 | 1.00 |  |  |  |
| ≥75 nmol/L |  | 25/30 | 0.83 | 0.45-1.55 | 0.56 |  | 23/28 | 0.73 | 0.37-1.43 | 0.36 |  |
| **Distal colon cancer** |  |  |  |  |  |  |  |  |  |  |  |
| Continuous (5 nmol/L increase) |  | 198/198 | 1.00 | 0.93-1.07 | 0.96 |  | 157/157 | 1.01 | 0.93-1.09 | 0.86 |  |
| *Grouped* |  |  |  |  |  |  |  |  |  |  |  |
| ≤30 nmol/L |  | 19/13 | 1.83 | 0.80-4.17 | 0.15 |  | 13/10 | 1.25 | 0.46-3.40 | 0.66 |  |
| >30 to <50 nmol/L |  | 72/72 | 1.23 | 0.75-2.02 | 0.41 |  | 57/53 | 1.52 | 0.85-2.74 | 0.16 |  |
| ≥50 to <75 nmol/L (reference value) |  | 82/96 | 1.00 |  |  |  | 67/81 | 1.00 |  |  |  |
| ≥75 nmol/L |  | 25/17 | 1.74 | 0.87-3.48 | 0.12 |  | 20/13 | 2.11 | 0.90-4.98 | 0.09 |  |
| **Rectal cancer** |  |  |  |  |  |  |  |  |  |  |  |
| Continuous (5 nmol/L increase) |  | 221/221 | 0.98 | 0.92-1.04 | 0.50 |  | 174/174 | 1.00 | 0.92-1.08 | 0.95 |  |
| *Grouped* |  |  |  |  |  |  |  |  |  |  |  |
| ≤30 nmol/L |  | 16/12 | 1.71 | 0.75-3.91 | 0.20 |  | 10/12 | 1.04 | 0.38-2.84 | 0.95 |  |
| >30 to <50 nmol/L |  | 86/76 | 1.43 | 0.91-2.24 | 0.12 |  | 69/58 | 1.55 | 0.89-2.69 | 0.12 |  |
| ≥50 to <75 nmol/L (reference value) |  | 90/111 | 1.00 |  |  |  | 72/88 | 1.00 |  |  |  |
| ≥75 nmol/L |  | 29/22 | 1.55 | 0.82-2.92 | 0.18 |  | 23/16 | 1.64 | 0.75-3.56 | 0.21 |  |
| *Adjusted by age, region of living and time of blood sampling (all through matching), with additional adjustments for physical activity, body mass index, smoking, processed meat, alcohol, calcium, and fiber intake | | | | | | | | | | |  |
|  |  |  |  |  |  |  |  |  |  |  |  |

**Supplementary Table 2** Sensitivity analysis of the risk of colorectal cancer, colon cancer, proximal colon cancer, distal colon cancer, and rectal cancer according to plasma 25-Hydroxyvitamin D levels. A nested case-control study (n=1550) from the Norwegian Women and Cancer post-genome cohort (2003-2006).

|  |  |  | Matched by age, region of living, and time of blood sampling | | |  | Multivariable analyses* | | |  |
| --- | --- | --- | --- | --- | --- | --- | --- | --- | --- | --- |
|  | n (cases / controls) |  | OR | 95% CI | *p* |  | OR | 95% CI | *p* |  |
| **Colorectal cancer** |  |  |  |  |  |  |  |  |  |  |
| *Grouped* |  |  |  |  |  |  |  |  |  |  |
| <50 nmol/L | 356/327 |  | 1.23 | 0.98-1.54 | 0.06 |  | 1.25 | 1.00-1.57 | 0.06 |  |
| ≥50 to <75 nmol/L (reference value) | 338/375 |  | 1.00 |  |  |  | 1.00 |  |  |  |
| ≥75 nmol/L | 81/73 |  | 1.22 | 0.85-1.74 | 0.35 |  | 1.19 | 0.82-1.73 | 0.35 |  |
| **Colon cancer** |  |  |  |  |  |  |  |  |  |  |
| *Grouped* |  |  |  |  |  |  |  |  |  |  |
| <50 nmol/L | 242/226 |  | 1.18 | 0.90-1.54 | 0.23 |  | 1.18 | 0.90-1.56 | 0.24 |  |
| ≥50 to <75 nmol/L (reference value) | 231/251 |  | 1.00 |  |  |  | 1.00 |  |  |  |
| ≥75 nmol/L | 52/48 |  | 1.17 | 0.75-1.84 | 0.49 |  | 1.16 | 0.73-1.84 | 0.52 |  |
| **Proximal colon cancer** |  |  |  |  |  |  |  |  |  |  |
| *Grouped* |  |  |  |  |  |  |  |  |  |  |
| <50 nmol/L | 149/137 |  | 1.15 | 0.83-1.60 | 0.40 |  | 1.19 | 0.84-1.68 | 0.32 |  |
| ≥50 to <75 nmol/L (reference value) | 147/154 |  | 1.00 |  |  |  | 1.00 |  |  |  |
| ≥75 nmol/L | 25/30 |  | 0.83 | 0.45-1.54 | 0.56 |  | 0.81 | 0.43-1.53 | 0.51 |  |
| **Distal colon cancer** |  |  |  |  |  |  |  |  |  |  |
| *Grouped* |  |  |  |  |  |  |  |  |  |  |
| <50 nmol/L | 91/85 |  | 1.31 | 0.81-2.11 | 0.28 |  | 1.27 | 0.76-2.11 | 0.36 |  |
| ≥50 to <75 nmol/L (reference value) | 82/96 |  | 1.00 |  |  |  | 1.00 |  |  |  |
| ≥75 nmol/L | 25/17 |  | 1.73 | 0.86-3.47 | 0.12 |  | 1.71 | 0.83-3.51 | 0.15 |  |
| **Rectal cancer** |  |  |  |  |  |  |  |  |  |  |
| *Grouped* |  |  |  |  |  |  |  |  |  |  |
| <50 nmol/L | 102/88 |  | 1.46 | 0.95-2.26 | 0.08 |  | 1.62 | 1.01-2.61 | 0.05 |  |
| ≥50 to <75 nmol/L (reference value) | 90/111 |  | 1.00 |  |  |  | 1.00 |  |  |  |
| ≥75 nmol/L | 29/22 |  | 1.55 | 0.82-2.92 | 0.17 |  | 1.62 | 0.82-3.20 | 0.17 |  |
| *Adjusted for age, region of living and time of blood sampling (all through matching), with additional adjustments for physical activity, body mass index, smoking, processed meat, alcohol, calcium, and fiber intake | | | | | | | | | |  |
|  |  |  |  |  |  |  |  |  |  |  |
